# Beliefs and attitudes of Syrian refugee mothers in Lebanon regarding children vaccination: a cross-sectional study

**DOI:** 10.1186/s12889-025-21290-w

**Published:** 2025-01-08

**Authors:** Issam Shaarani, Sleiman Khadem, Marwa Obeid, Behnaz Saadieh, Aya Serhal, Karim Zakkour, Sara Mohammad, Hussein Berjaoui, Nour Izmirli

**Affiliations:** Faculty of Medicine, Beirut Arab University, Beirut, Lebanon

**Keywords:** Vaccination, Syrian refugees, Immunization

## Abstract

**Background:**

Vaccines have contributed to the disappearance of various diseases, and almost eradicated others across the world. Studies have shown that in Lebanon a profoundly small percentage of Syrian refugee children were fully immunized by routine vaccination services. Exploring the knowledge, attitudes, and practices of parents towards vaccination is of crucial importance, given the role of parents in children’s immunization. This study aims to investigate the knowledge, attitudes, and practices of the Syrian refugee mothers in Lebanon towards the immunization of their children.

**Methods:**

This is a cross-sectional study conducted using questionnaires targeting Syrian refugee mothers whose children were born on Lebanese grounds, are below the age of five, and are following the Lebanese vaccination schedule.

**Results:**

Majority of refugee mothers considered vaccination to be safe (89.9%) and stated that vaccination should be initiated at birth (87.2%). Almost all of the interviewed mothers plan to vaccinate their children according to the National Lebanese Vaccination Schedule. Concerning the children’s immunization status, 51.4% of Syrian refugee children were fully immunized and 48.6% had aberrant vaccination.

**Conclusion:**

Although refugee mothers had some knowledge gaps regarding vaccines, the main issue lies within the accessibility. A collaborative coordinated approach involving governmental and non-governmental agencies seems to be an effective approach to improve rates of immunization.

**Supplementary Information:**

The online version contains supplementary material available at 10.1186/s12889-025-21290-w.

## Background

Amongst the various primary prevention methods used in the public health sector, vaccination is regarded as one of its most highlighted achievements [1]. Vaccination’s importance and effectiveness have been proven by its ability to reduce death and morbidity due to infectious diseases worldwide, saving 2 to 3 million lives yearly [2]. In fact, its benefit is not only limited to those vaccinated but also grants herd immunity to those who are not, which further limits the spread of communicable diseases [2]. Vaccines have contributed to the disappearance of various diseases (i.e. smallpox), and almost eradicated others (i.e. polio) across the world. It also helped decrease infections caused by many viruses and bacteria in most of the developed countries, granted by their advanced healthcare system [3]. Despite this evolution in the healthcare systems worldwide, about 19.4 million children, predominantly among refugees and people in areas of conflict, remain unimmunized and suffer from high morbidity and mortality from vaccine-preventable diseases, as they have restricted access to vaccination [4, 5]. It is unfortunate that almost half of the pediatric deaths are accounted for preventable communicable diseases [6].

Since 2011, the situation in Syria has been deteriorating causing the displacement of many Syrian citizens to the neighboring countries such as Lebanon [7]. According to UNHCR, a total of 976,000 Syrian refugees were present in Lebanon up till 31st July 2018 [8], and that constitutes about 30% of the total population [9]. The Syrian conflict has caused constant displacement of refugees mostly to areas that were already crowded and lacked proper sanitation. This has led to outbreaks of infections across Syria and the neighboring countries, damaging its healthcare system and causing loss of information concerning their vaccination status; more than 50% of the 1.8 million children born since this conflict couldn’t get access to vaccination [10–12]. A study has shown that in Lebanon only 12.5% of Syrian refugee children were fully immunized by the routine vaccination services. These numbers are quite low compared to the 46.6% of the children in the Lebanese households. Thus, the importance of vaccination among displaced populations must be addressed given their intangible human right for healthcare, including vaccination [10, 11]. Exploring the knowledge, attitudes and practices of parents towards vaccination is of crucial importance given the role of parents in children’s immunization [13]. The factors that influence these variables should also be taken into consideration [14]. For instance, many parents exhibit vaccine hesitancy as they do not believe in the advantages of vaccines and doubt their extent of safety, all while assuming that vaccination is not crucial for their child. Some parents also fear the side effects that vaccines can cause, as they believe that side effects would be more serious than the preventable disease itself [14].

To complete the picture on immunization among Syrian refugee children below five years of age, this study aims to investigate the knowledge, attitudes, and practices of the Syrian refugee mothers in Lebanon towards the immunization of their children. The primary hypothesis of this study is that the immunization rate among Syrian refugee children in Lebanon is suboptimal. We further hypothesize that this low rate is associated with specific maternal factors, including sociodemographic characteristics, and the presence of knowledge gaps or reservations regarding childhood vaccination.

## Methods

### Study design, area, and participants

This is a cross-sectional study conducted through questionnaires that were distributed in four different regions in Lebanon where Syrian refugees mostly reside: Beirut, Bekaa, South, and North Lebanon. The participants were Syrian refugee mothers whose children were born on Lebanese grounds, are below the age of five, and are following the Lebanese vaccination schedule.

The participants were approached during their visits to pediatricians or family physicians at different healthcare centers serving mostly Syrian refugees including Tahaddi and Ibad Al-Rahman healthcare centers in Beirut, Ghiras Al Khair and Barelias medical centers in Bekaa, Serepta and Al- Bunyan healthcare centers in South Lebanon, and Al-Iman and Al Qubba Islamic (Ibn Sina) healthcare centers in North Lebanon.

### Sample size and data collection

The questionnaire was designed in English and translated into Arabic by the investigators (Appendix 1). It included questions on sociological and demographical characteristics of the study population, questions assessing knowledge, attitudes, and practices of mothers regarding immunization, and questions regarding vaccine uptake according to the National Lebanese Vaccination Schedule (Appendix 2) [15].

A pilot study on 20 participants was conducted before starting the data collection. To ensure cultural and contextual relevance, the questionnaire was reviewed by healthcare professionals with experience in the Syrian refugee context and then pilot-tested with a small group of Syrian refugee mothers. Feedback from the pilot helped us refine questions to improve clarity and appropriateness for the target population.

Data collection took place from July 2018 to January 2019. Convenient sampling was used to select the participants who were stratified by governorate. The sample size calculation was based on a power of 80% and a significance level of 0.05, estimating that a sample of 384 participants would provide sufficient power to detect the proportion of fully immunized children. To account for incomplete responses of 20%, we set the target sample size at 480.”

Due to the unequal distribution of Syrian refugees across different regions in Lebanon, we employed a stratified sampling technique. We determined the sample size for each governorate based on the proportion of the refugee population residing in that region, ensuring representation across Beirut, Bekaa, South Lebanon, and North Lebanon. This proportional allocation allowed us to reflect the regional diversity within the Syrian refugee population in our sample. According to UNHCR, a total of 976,000 Syrian refugees were present in Lebanon up till 31 July 2018. The distributions according to regions were as follows: 254,993 in Beirut, 351,972 in Bekaa, 117,500 in South and 251,537 in North Lebanon [7]. Thus, a total of 126 participants were included from Beirut, 175 from Bekaa, 58 from South and 125 from North Lebanon.

During the interview, the mothers were asked to present their child’s vaccination card, if accessible, for the assessment of their immunization status. For mothers who did not have the vaccination card at the time of the interview, we asked about each vaccine their child received and documented completeness based on their responses. Among the participants, 226 mothers had the vaccination card during the interview, 232 reported having it at home, and 24 did not possess a vaccination card at all.

To ensure accuracy, we compared the proportion of fully immunized children between those who had a vaccination card present and those who did not. We found no significant difference in immunization rates between these groups. Additionally, the associations between immunization completion rates and sociodemographic variables remained consistent, regardless of whether a vaccination card was available.

### Statistical analysis

SPSS 23 was used for analysis. For descriptive purposes, continuous variables were displayed as means ± standard deviations. Categorical variables were presented as numbers and percentages. A chi-square test for independence was employed to evaluate the associations between categorical variables, including sociodemographic characteristics and immunization status.

## Results

### Sample characteristics and immunization status

A total of 484 Syrian refugee mothers participated in this study. Among these, 51.4% reported that their children were fully immunized according to their age. The age of the interviewed Syrian refugee mothers ranged from 15 to 47 years (mean age of 26.73 ± 5.97 years). Regarding the educational level, the highest percentage recorded (31%) was for mothers reaching secondary school. The majority of the participants were unemployed (90.9%). Neither education nor employment status was associated with vaccination status of the respective children. The residency of the mother was significantly associated with the child immunization status (*p*-value = 0.002) in a way where those who lived in an apartment/house had the highest percentage of children vaccinated (64.3%).As for the regions in which the Syrian refugees were distributed, those who resided in Beirut had the highest percentage for children who were fully immunized (63.5%), with a significance of 0.019. The age of children of interviewed mothers ranged from 1 to 58 months (mean age of 20.23 ± 15 months). Other characteristics regarding the demographics of the sampled mothers are recorded in Table 1.

**Table 1:** Association between sociodemographic characteristics and immunization status

Characteristics	*N*	% Of Fully Immunized Children	*P*-value
*Age of mother*			
Mean ± SD	26.73 ± 5.97	51.4	0.98
*Age of child*			
Mean ± SD	20.23 ± 15	51.4	0.993
*Educational Status of mother*			
No formal education	86 (17.8)	54.7	0.071
Primary school	141 (29.1)	41.1	
Secondary School	154 (31.8)	55.2	
High school	70 (14.5)	57.1	
College	33 (6.8)	57.6	
*Current Residence*			
Apartment/House	284 (58.7)	56.3	**0.010**
Others*	200 (41.3)	44.5	
*Occupation of mother*			
Employed**	44 (9.1)	47.7	0.605
Unemployed	440 (90.9)	51.8	
*Gender of the child*			
Male	260 (53.7)	47.7	0.075
Female	224 (46.3)	55.8	
*Complete Vaccination according to the region*			
Beirut	126 (26.0)	63.5	**0.019**
North Lebanon	125 (25.8)	48.0	
South Lebanon	58 (12.0)	46.6	
Bekaa	175 (36.2)	46.9	
*Order of child among siblings*			
First born	136 (28.1)	53.7	0.539
Not first born	348 (71.9)	50.6	

* Unfinished building, room, camp, garage, construction site/ work place, compounds, and others

**Housekeeper, secretary, farmer, and others

### Knowledge of childhood immunizations

As shown in Table 2, most respondents (79.8%) stated that the purpose of vaccination was the prevention of vaccine-preventable diseases but also many (73.3%) thought that vaccines can prevent non-communicable diseases as well. Over half of the participants (54.8%) interviewed thought that vaccines actually treat diseases. As for diseases that could be prevented by vaccination, polio and measles recorded the highest percentages with 85.5% and 87.2%, respectively. It was also noted that hepatitis B recorded the highest percentage (74.9%) for a fatal disease that was believed to necessitate vaccination. Around 23% of mothers had the perception of diabetes mellitus being one of the vaccine preventable diseases. There is no statistically significant difference between mothers of immunized and never-immunized children when it comes to knowledge regarding purpose of vaccination.

The majority of the mothers considered vaccinations to be safe (89.9%) and 87.2% stated that vaccination should be initiated at birth. Around 65% of the mothers stated that vaccination causes side effects, most of which were pain at the site of injection (79.2%) and mild fever (97.4%). Most mothers stated that otitis media (74.6%) and common cold (75.4%) were contraindications to vaccination. There is no significant difference between the immunization status of the child and the belief that vaccination adverse effects are dangerous.

43.4% of mothers with fully immunized children do search around for information about immunization versus 31.1% of those who have incomplete or absent immunization. This difference is statistically significant (*P* = 0.009).

**Table 2:** Mothers’ knowledge regarding child’s immunization

Characteristics	*N* (%)
*Perception of their immunization knowledge*	
Good	282 (58.3)
Fair	174 (36.0)
Poor	28 (5.8)
*Purpose of vaccination*	
Treatment of disease	265 (54.8)
Prevention of non-communicable diseases	355 (73.3)
Prevention of communicable diseases	386 (79.8)
Permission of child development	261 (53.9)
Maintenance of good health	303 (62.6)
I do not know	22 (4.5)
*Vaccine initiation*	
At birth	422 (87.2)
At 6 weeks	25 (5.2)
Anytime	37 (7.6)
*Safety of vaccination*	
Safe	435 (89.9)
Somewhat safe	48 (9.9)
Unsafe	1 (0.2)
*Diseases prevented by vaccination*	
Polio	414 (85.5)
Measles	422 (87.2)
Jaundice	288 (59.5)
Common cold	160 (33.1)
Hepatitis B	332 (68.6)
Diphtheria	155 (32.0)
Diabetes	109 (22.5)
Asthma	154 (31.8)
Pertussis	223 (46.1)
Tetanus	290 (59.9)
Fatal diseases that necessitate vaccination*	
Polio	256 (59.0)
Measles	271 (61.5)
Jaundice	180(52.9)
Common cold	54(22.5)
Hepatitis B	277 (74.9)
Diphtheria	85 (37.9)
Diabetes	71 (37.0)
Asthma	89 (39.9)
Pertussis	117 (41.8)
Tetanus	168 (49.7)
*Presence of side effects*	312 (64.5)
Mild fever	304 (97.4)
Pain at site of injection	247 (79.2)
Rash/redness	105 (33.7)
Body weakness	108 (34.6)
Body swelling	86 (27.6)
Shivering	37 (11.9)
Headache	28 (9.0)
Diarrhea	59 (18.9)
Others	4 (1.3)
Are they dangerous?	21 (6.7)
*Consequences of vaccination*	
Chronic diseases	11 (2.3)
Autism	5 (1.0)
*Contraindications to vaccination*	
Otitis media	361 (74.6)
Common cold	364 (75.4)
Diarrhea	338 (69.8)
Antibiotics	343 (70.9)
*Harmful consequences of multiple vaccination*	161 (33.3)

*This question was answered by mothers who answered “yes” in the previous question

### Attitudes towards childhood immunizations

When asked whether the mothers were in favor of vaccination, about 98.0% responded that they were. Of mothers who search for information regarding immunization, around half revealed that they rely on healthcare providers. Almost all of the interviewed mothers plan to vaccinate their children according to the National Lebanese Vaccination Schedule. Table 3 further details more information about the attitudes of the interviewed mothers.

**Table 3:** Attitudes of mothers towards vaccination

Characteristics	*N* (%)
*Favors vaccination*	474 (97.9)
*Searches for information about vaccination*	190 (39.3)
*Searches for information about vaccination*	190 (39.3)
Internet	80 (42.1)
Healthcare providers	94 (49.5)
Neighbors	21 (11.1)
Others*	18 (9.4)
*Planning to fully vaccinate children according to Lebanese schedule*	478 (99.0)
*Interested in having more information about vaccination*	421 (87.0)

*Family, friends, and TV

### Practices of mothers regarding childhood immunizations

Of the interviewed mothers, 95% said that their child has an immunization card. Doctors were considered the main source of information to mothers (79.1%) regarding the immunization of children. Most of the participants initiated the vaccination of their children at birth. Among those who mentioned that side effects occurred once vaccinations were given (30.6%), the most common side effect reported was mild fever (27.9%) which they mostly chose to relieve by administration of anti-pyrectics. The minority could recall the next or last vaccine that their child needs to take or have taken. Further information regarding the practices of mothers is listed in Table 4.

**Table 4:** Practices of mothers regarding vaccination

Characteristics	*N* (%)
*Child with immunization card*	460 (95.0)
*Both parents as decision makers for child immunization*	333 (68.8)
*Sources of information regarding immunization*	
Doctor	383 (79.1)
Nurse	112 (23.1)
Pharmacist	44 (9.1)
Campaigns	101 (20.9)
Social media	50 (10.3)
Television	47 (9.7)
Friends	75 (15.5)
Family	157 (32.4)
Others*	46 (9.5)
*Vaccine initiation at birth*	427 (91.0)
*Ways used to eliminate the side effects if present*	
Give anti-pyretic	123 (25.4)
Give extra fluids	22 (4.5)
Put cold and wet clothes	17 (3.5)
Others**	22 (4.5)
*Informing workers about the side effects and their response*	49 (33.1)
*Rejection of a non-halal vaccine*	104 (21.5)
*Can recall the Next vaccination child must get*	47 (9.7)
*Can recall the Last vaccination child received*	74 (15.3)

* Radio, newspaper, and others

**Bathe in cool water, overdress, give antibiotics

## Discussion

This manuscript explores the knowledge, attitudes, and practices regarding childhood immunization among Syrian refugee mothers in Lebanon and sheds light on their association with sociodemographic factors.

The demographic characteristics emphasize the challenges faced by Syrian refugee mothers, with a considerable proportion being unemployed and having a primary education level of secondary school. Literature discusses the potential reasons leading to low vaccination rates, including parental, recipient, and vaccination-process factors. For parental factors, lower vaccination rates were previously noted among parents, especially mothers, with low or no academic education, along with those who are young of age. Both conditions could signify a lack of awareness regarding vaccines and their efficiency which negatively affects vaccination [6, 9, 16]. However, in our study, there was no difference in vaccination rates of one’s child when it comes to educational or work status of the mother, including having no formal education (*p* = 0.071), being unemployed (*p* = 0.605), or even the age of the mother (*p* = 0.98). This might be indicative of potential presence of confounding barriers to healthcare access or other socio-economic challenges that go beyond the conventional predictors of immunization behavior.

The place of residence seems to be associated with the immunization status, with mothers residing in apartments or houses displaying a higher percentage of fully immunized children. Our data shows that only 48% and 46.9% of refugee children settling in North and Bekaa, respectively, were immunized. This suggests a potential link between living conditions and access to healthcare services. Coinciding with the Syrian refugee’s influx to Lebanon, there was an increase in the hepatitis A cases up to 2–3 folds, along with other infectious diseases like measles and mumps around 3–5 folds, especially in Beqaa and North governorates settlements [9, 17]. A surge in infectious diseases among refugees has also been witnessed in other countries receiving refugees, and that is expected as the preventive services and crowding control measures inside refugee camps are suboptimal [18, 19]. Indeed, the crowding and the lack of sanitary infrastructure in refugee camps makes infection transmission more likely [10]. Further investigation into factors contributing to this association could reveal critical insights for targeted interventions.

As for factors concerning the child, while the child’s gender did not affect vaccination rates [16], lack of continuous follow-up with a pediatrician, a coinciding illness at the time of vaccination, as well as a child’s age (*p* < 0.001) all played a significant role and match up with the information we have from previous studies [6, 9, 16]. In our study, infants up to 6 months of age had more adequate patterns of vaccinations compared to older children between 6 months and 5 years of age, coinciding with literature [9].

Approximately 48.6% of the children in our study exhibited aberrant vaccination patterns, indicating either partial or complete lack of immunization, whereas data collected in 2015 showed that 79.9% of Syrian refugee children in Lebanon had incomplete vaccination [20]. Wide efforts have been made by the Lebanese MOPH in collaboration with UNICEF and WHO within this timeline to reduce the occurrence of vaccine-preventable diseases to the least possible by ensuring timely and complete access to vaccination for the entire population, focusing on the most vulnerable [21]. The difference in data between this study and data in 2015 presumes the successful contribution of these interventions in the improvement of vaccinations rates, even amidst several national health and economic crises. Despite minor knowledge gaps among refugee mothers, targeting health education, raising awareness among healthcare workers about their vital role, improving provider communication, and providing Arabic informational materials may further support vaccination efforts among the studied population.

In Syria, vaccination rates dropped to almost half by 2013 in some areas due to the ongoing war, especially for diseases like diphtheria, tetanus and pertussis, as they are considered to be the main vaccine-preventable ones [4, 10]. 33.9% of the children in our study were either partially or never immunized. It seems that partially vaccinating children with a single dose is a common trend [16, 22], but this could also be due to missed vaccinations as a consequence of the ongoing crisis. Vaccination catch-up should be structured to replenish the missed immunity, especially if the vaccination schedules among host and origin country differ [19]. Noteworthy is the acknowledgment of the widespread impact of the SARS-CoV-2 pandemic-which occurred later-on the global immunization rates. This reality is highlighted by a study conducted in Romania, revealing a significant decline in MMR and hexavalent vaccination rates to below 60% and 70%, respectively, during the pandemic [23]. This emphasizes the importance of ongoing research to direct and respond effectively to the dynamic factors influencing childhood immunization in the wake of global health crises.

In comparison, Lebanese children have higher prevalence of vaccination even though many vaccination campaigns were organized to target Syrian population since the refugee’s influx [16]. Unfortunately, there are limited capacities for the health sector in Lebanon to cover the needs of the increasing number of refugees and accompanied outbreaks despite virtually all mothers participating in our study planning to vaccinate their children according to the National Lebanese Vaccination Schedule [9, 16]. It has been proposed that the medical needs of these refugees were never adequate [7], and they themselves believe their health status in general is below the required level, mainly due to the high costs [24]. Healthcare system in Lebanon is mostly dominated by private institutions, leaving hardship for access by refugees [12]. The negative impact of this situation can be seen through the low compliance to non-obligatory vaccines versus obligatory ones (approximately 20 vs. 40%) [9]. This difference can be accounted to the price of the non-mandatory vaccines, compared to the mandatory counterpart which are free by the Ministry of Public Health [9]. The obligatory vaccines (Appendix 1) are those mandated by the Lebanese ministry of Public Health and every child living on the Lebanese grounds must receive them [15]. Said vaccines are readily available for free in dispensaries across all the districts. Non-mandatory vaccines, while often still available at the dispensary of healthcare centers, had a fee ranging from 55 US dollars to 70 US dollars, which likely affected the willingness to vaccinate. There were some attempts to provide some degree of free healthcare, such as the polio vaccination campaigns organized in several schools and camps starting November 2013 [25]. This study assessed general attitudes toward vaccination without differentiating between vaccine hesitancy and specific concerns about particular vaccines. Future research could explore this distinction to gain deeper insight into vaccine-specific attitudes among Syrian refugee mothers. The knowledge assessment reveals a generally good understanding of vaccination purposes among the respondents. However, the perception of non-communicable diseases being preventable by vaccines and the belief that vaccines can treat diseases highlight areas where targeted education campaigns may be beneficial. In a study about immunization knowledge in Nigeria, some women did not even know the purpose of vaccinating children, and this lack of knowledge, along with a low level of education, is found to be the main culprit of low vaccination rates [26].

The overwhelmingly positive attitude towards vaccination, with 98.0% of mothers expressing favorability, aligns with global trends supporting immunization. The reliance on healthcare providers for information underscores the pivotal role of these professionals in shaping parental attitudes. Strengthening this patient-physician relationship could further enhance vaccination advocacy.

Vaccination was considered safe among Syrian refugees in Lebanon, as only 10.1% had safety concerns. In comparison, a cross sectional study done in 2022 revealed that the fear of vaccine adverse reactions represented 26.6% of reasons causing Romanian parents to decline their children’s vaccination [27]. More studies need to investigate the reasons behind such reasons for hesitancy and different rates among populations. The finding that a considerable proportion of mothers considered otitis media and the common cold as contraindications to vaccination is intriguing. This misconception could be addressed through focused health education initiatives to clarify the distinctions between contraindications and common side effects, potentially mitigating vaccine hesitancy. As for one of the misconceptions that trended among parents in the past decade, only 1% of refugee mothers believed vaccines could possibly cause autism consequently.

### Limitations

This study did not assess parental vaccination status. The literature makes a valid point regarding parental tolerance to vaccination; they are more accepting if they, themselves, had taken the vaccines previously and with no side effects [6]. Moreover, due to accessibility, we have not included the fathers’ educational level and knowledge about vaccination into analysis. Finally, the use of convenient sampling in this survey introduces a risk of selection bias, potentially affecting the representativeness of the results. Future research employing more rigorous methods, like random sampling, could reduce these biases and provide a clearer understanding of Syrian refugee mothers’ views on children’s vaccination.

## Conclusion

It appears to be that the knowledge of Syrian refugees’ mothers towards vaccination and its side effects is not the major issue when it comes to vaccinating their children. However, the issue lies with the inaccessibility of vaccines for the population. It is obvious that the Syrian refugees’ mothers seek to validate their information and strengthen their knowledge in the topic of immunization from reliable resources.

The outcome of this study emphasizes the importance of addressing both knowledge gaps and access barriers in vaccination programs, particularly among vulnerable populations.

Finally, the results highlight the value of collaborative approach and coordination between governmental and non-governmental agencies in times of crises, particularly when it comes to the provision of essential health services like immunization.

## Electronic supplementary material

Below is the link to the electronic supplementary material.

Supplementary Material 1

Supplementary Material 2

## Data Availability

The data that support the findings of this study are available from the corresponding author upon reasonable request.

## References

[1] Ames HM, Glenton C, Lewin S. Parents’ and informal caregivers’ views and experiences of communication about routine childhood vaccination: a synthesis of qualitative evidence. Cochrane Database Syst Reviews. 2017(2). 10.1002/14651858.CD011787.pub2 PMC5461870 28169420

[2] Skinner E. Vaccination policies: requirements and exemptions for entering School. NCSL Legisbrief. 2017;25(48):1–2. 29320811

[3] Delany I, Rappuoli R, De Gregorio E. Vaccines for the 21st century. EMBO Mol Med. 2014;6(6):708–20. 24803000 10.1002/emmm.201403876 PMC4203350

[4] Nnadi C, Etsano A, Uba B, Ohuabunwo C, Melton M, wa Nganda G, et al. Approaches to vaccination among populations in areas of conflict. J Infect Dis. 2017;216(suppl1):S368–72. 28838202 10.1093/infdis/jix175 PMC5754212

[5] Khader YS, Laflamme L, Schmid D, El-Halabi S, Khdair MA, Sengoelge M, et al. Children immunization app (CImA) among Syrian refugees in Zaatari camp, Jordan: protocol for a cluster randomized controlled pilot trial intervention study. JMIR Res Protocols. 2019;8(10):e13557. 10.2196/13557 PMC6803890 31593549

[6] Smith LE, Amlôt R, Weinman J, Yiend J, Rubin GJ. A systematic review of factors affecting vaccine uptake in young children. Vaccine. 2017;35(45):6059–69. 28974409 10.1016/j.vaccine.2017.09.046

[7] El-Khatib Z, Scales D, Vearey J, Forsberg BC. Syrian refugees, between rocky crisis in Syria and hard inaccessibility to healthcare services in Lebanon and Jordan. Confl Health. 2013;7(1):1–3. 24004474 10.1186/1752-1505-7-18 PMC3848432

[8] UNHRC. Operational Data Portal. Syria Regional Refugee Response in Lebanon [Available from: https://data2.unhcr.org/en/situations/syria/location/71

[9] Kmeid M, Azouri H, Aaraj R, Bechara E, Antonios D. Vaccine coverage for Lebanese citizens and Syrian refugees in Lebanon. Int Health. 2019;11(6):568–79. 31089721 10.1093/inthealth/ihz023

[10] Sharara SL, Kanj SS. War and infectious diseases: challenges of the Syrian civil war. PLoS Pathog. 2014;10(11):e1004438. 25393545 10.1371/journal.ppat.1004438 PMC4231133

[11] Roberton T, Weiss W, Doocy S, Team JHAS, Team LHAS. Challenges in estimating vaccine coverage in refugee and displaced populations: results from household surveys in Jordan and Lebanon. Vaccines. 2017;5(3):22. 28805672 10.3390/vaccines5030022 PMC5620553

[12] Ozaras R, Leblebicioglu H, Sunbul M, Tabak F, Balkan II, Yemisen M, et al. The Syrian conflict and infectious diseases. Expert Rev anti-infective Therapy. 2016;14(6):547–55. 27063349 10.1080/14787210.2016.1177457

[13] Šeškutė M, Tamulevičienė E, Levinienė G. Knowledge and attitudes of postpartum mothers towards immunization of their children in a Lithuanian tertiary teaching hospital. Medicina. 2018;54(1):2. 30344233 10.3390/medicina54010002 PMC6037235

[14] Yaqub O, Castle-Clarke S, Sevdalis N, Chataway J. Attitudes to vaccination: a critical review. Soc Sci Med. 2014;112:1–11. 24788111 10.1016/j.socscimed.2014.04.018

[15] MOPH. National Immunization Calendar. 2016 [Available from: https://www.moph.gov.lb/userfiles/files/HealthCareSystem/EPI/NationalCalendarforVaccination.pdf

[16] Mansour Z, Hamadeh R, Rady A, Danovaro-Holliday MC, Fahmy K, Said R, et al. Vaccination coverage in Lebanon following the Syrian crisis: results from the district-based immunization coverage evaluation survey 2016. BMC Public Health. 2019;19(1):1–11. 30642314 10.1186/s12889-019-6418-9 PMC6332691

[17] Bizri AR, Fares J, Musharrafieh U. Infectious diseases in the era of refugees: Hepatitis a outbreak in Lebanon. Avicenna J Med. 2018;8(4):147. 30319956 10.4103/ajm.AJM_130_18 PMC6178566

[18] Tayfur I, Günaydin M, Suner S. Healthcare service access and utilization among Syrian refugees in Turkey. Annals Global Health. 2019;85(1). 10.5334/aogh.2353 PMC6634327 30896133

[19] Lam E, McCarthy A, Brennan M. Vaccine-preventable diseases in humanitarian emergencies among refugee and internally-displaced populations. Hum Vaccines Immunotherapeutics. 2015;11(11):2627–36. 10.1080/21645515.2015.1096457 PMC4685677 26406333

[20] Roberton T, Weiss W, Doocy S. Challenges in estimating Vaccine Coverage in Refugee and displaced populations: results from Household surveys in Jordan and Lebanon. Vaccines (Basel). 2017;5(3). 10.3390/vaccines5030022 PMC5620553 28805672

[21] MOPH. National Immunization Strategy 2017–2022.

[22] Doocy S, Lyles E, Roberton T, Weiss W, Burnham G. Health access and utilization survey among non-Camp Syrian refugees in Jordan. 2014. 2020.

[23] Herdea V, Ghionaru R, Lungu CN, Leibovitz E, Diaconescu S. Vaccine Coverage in Children Younger Than 1 Year of Age during Periods of High Epidemiological Risk: Are We Preparing for New Outbreaks? Children (Basel, Switzerland). 2022;9(9). 10.3390/children9091334 PMC9497469 36138643

[24] Strong J, Varady C, Chahda N, Doocy S, Burnham G. Health status and health needs of older refugees from Syria in Lebanon. Confl Health. 2015;9(1):1–10. 26056531 10.1186/s13031-014-0029-y PMC4459463

[25] Alawieh A, Sabra Z, Langley EF, Bizri AR, Hamadeh R, Zaraket FA. Assessing the impact of the Lebanese National Polio Immunization Campaign using a population-based computational model. BMC Public Health. 2017;17(1):1–11. 29178859 10.1186/s12889-017-4909-0 PMC5702188

[26] Tagbo B, Uleanya N, Omotowo I. Mothers’ knowledge and perception of adverse events following immunization in Enugu, Southeast Nigeria. J Vaccines Vaccin. 2013;4(202):2.

[27] Miron VD, Toma AR, Filimon C, Bar G, Craiu M. Optional Vaccines in Children—Knowledge,Attitudes, and Practices in Romanian Parents. Vaccines. 2022;10(3):404. 10.3390/vaccines10030404 PMC8955643 35335036

