# Supplementary material for: Beliefs and attitudes of Syrian refugee mothers in Lebanon regarding children vaccination: a cross-sectional study
Source: BMC Public Health. 2025 Jan 8;25:99. doi: 10.1186/s12889-025-21290-w (PMC11715023; doi:10.1186/s12889-025-21290-w)
Supplement: Supplementary file 1 — Supplementary Material 1 [file 12889_2025_21290_MOESM1_ESM.pdf]

Appendix 2: National Lebanese Vaccination Schedule.

**Age**	**Vaccine**
At birth	Hepatitis B – First dose
2 months	IPV[+]– First dose<br>Pentavalent (diphtheria, pertussis, tetanus, hepatitis B, Hib) – First dose
4 months	OPV[++] – Second dose<br>Pentavalent (diphtheria, pertussis, tetanus, hepatitis B, Hib) – Second dose<br>PCV13[x] – First dose
6 months	OPV[++] – Third dose<br>Pentavalent (diphtheria, pertussis, tetanus, hepatitis B, Hib) – Third dose<br>PCV13[x] – Second dose
9 months	Measles – Zero dose
12 months	MMR[†] – First dose<br>PCV13[x] – First booster dose
18 months	OPV[++] – First booster dose<br>Pentavalent (diphtheria, pertussis, tetanus, hepatitis B, Hib) – First booster dose<br>MMR[†] – Second dose
4-5 years	OPV[++] – Second booster dose<br>DTP[§]

[+]Inactivated poliovirus vaccine
[++]Oral poliovirus vaccine
[x] Pneumococcal conjugate vaccine 13
† Measles, Mumps, and Rubella
[§] Diphtheria, Tetanus, Pertussis
