# Supplementary material for: Beliefs and attitudes of Syrian refugee mothers in Lebanon regarding children vaccination: a cross-sectional study
Source: BMC Public Health. 2025 Jan 8;25:99. doi: 10.1186/s12889-025-21290-w (PMC11715023; doi:10.1186/s12889-025-21290-w)
Supplement: Supplementary file 2 — Supplementary Material 2 [file 12889_2025_21290_MOESM2_ESM.pdf]

Appendix 1 – Questionnaire

Immunization Patterns of Children Aged up to Five Years Born to Refugee Mothers in Lebanon

**A. Sociological and demographic characteristics of the study population.**	
I. Mother's Characteristics:	
1. Age	
2. Educational status	1. No formal education<br>2. Primary school<br>3. Secondary school<br>4. High school<br>5. College
3. Number of children	
4. Current Residence	1. Apartment/House<br>2. Unfinished building<br>3. Camp<br>4. Unused garage<br>5. Construction site/ Work place<br>6. Compounds<br>7. Others(specify): ____
5. Do you work	1.Yes<br>**2. No** ——> **Go to question #7**
6. What's your occupation	1. Farmer<br>2. Secretary<br>3. Housekeeper<br>4. Other(specify): ____
II. Child Characteristics:	
7. Gender	1. Male<br>2. Female
8. Date of birth	
9. First born	**1.Yes** ——> **Go to question # 11**<br>2.No
10. Please specify:	
Past Medical History:	
11. Does your child suffer from a major medical illness?	1.Yes<br>**2.No** ——> **Go to question # 13**
12. Please specify:	

Appendix 1 – Questionnaire

13. Did your child undergo any previous surgeries?	1.Yes<br>2.No
14. Do you give your child any current medications?	1. Yes<br>**2. No** ——> **Go to question # 16**
15. Specify:	
16. Is your child known to have any allergies?	1.Yes<br>**2.No** ——> **Go to question # 18**
17. Please specify:	
Birth History	
18. Gestational age at delivery	1. Mature<br>2. Premature
Neonatal period	
19. Birth weight	
20. Did your child suffer from problems during his/her neo-natal period?	1.Yes<br>2.No
21. Was your child admitted to the hospital during this period?	1.Yes<br>2.**No** ——> **Go to question # 23**
22. Why was he admitted to the hospital?	
23. Did your child suffer from any illness?	1. Yes<br>**2. No** ——> **Go to question # 25**
24. Please specify;	
**B. Assessing the knowledge of mothers regarding immunization**	
25. What is the purpose of vaccination? (Choose all that apply)	1. Treat diseases<br>2. Prevent non-communicable diseases<br>3. Prevent communicable diseases<br>4. Grant child development<br>5. Maintain good health<br>6. I don't know
26. When should immunization be initiated?	1. At birth<br>2. At 6 weeks<br>3. Anytime

Appendix 1 – Questionnaire

27. How safe do you think immunizations are for children?	1. Safe<br>2. Somewhat safe<br>3. Unsafe
28. Do you believe that side effects occur with vaccination?	1.Yes<br>**2.No** ——> **Go to question # 31**<br>3.Sometimes
29. Are the side effects dangerous?	1.Yes<br>2.No<br>3.I don't know
30. Do you think vaccines cause:	
• Mild Fever	1.Yes<br>2.No<br>3.I don't know
• Pain at site of injection	1.Yes<br>2.No<br>3.I don't know
• Rash/Redness	1.Yes<br>2.No<br>3.I don't know
• Body weakness	1.Yes<br>2.No<br>3.I don't know
• Body swelling	1.Yes<br>2.No<br>3.I don't know
• Shivering	1.Yes<br>2.No<br>3.I don't know
• Headache	1.Yes<br>2.No<br>3.I don't know
• Diarrhea	1.Yes<br>2.No<br>3.I don't know
• Others(specify):	______________________
31. Do you believe that vaccination can cause chronic diseases?	1. Yes<br>2. No<br>3. I don't know

Appendix 1 – Questionnaire

32. Do you believe that vaccination may lead to autism?	1.Yes<br>2.No<br>3.I don't know
33. Does giving more than one vaccine at a time to the child harm his/her immunity?	1.Yes<br>2.No<br>3.I don't know
34. Can a child be vaccinated if he/she is currently taking an antibiotic?	1.Yes<br>2.No<br>3.I don't know
35. Can a child be vaccinated if he/she is currently suffering from otitis media?	1.Yes<br>2.No<br>3.I don't know
36. Can a child be vaccinated if he/she is currently suffering from common cold?	1.Yes<br>2.No<br>3.I don't know
37. Can a child be vaccinated if he/she is currently suffering from diarrhea?	1.Yes<br>2.No<br>3.I don't know
38. Which of the following diseases can be prevented by vaccination? (Choose all that apply)	1. Polio 6.Diphtheria<br>2. Measles 7.Diabetes<br>3. Jaundice 8.Asthma<br>4. Common cold 9.Pertussis<br>5. Hepatitis B 10.Tetanus
39. Which of the following diseases are fatal so that the child must be immunized against? (Choose all that apply)	1. Polio 6.Diphtheria<br>2. Measles 7.Diabetes<br>3. Jaundice 8.Asthma<br>4. Common cold 9.Pertussis<br>5. Hepatitis B 10.Tetanus
**C. Assessment of mothers attitude towards immunization**	
40. Are you in favor of vaccination?	**1.Yes** ——> **Go to question # 42**<br>2.No<br>**3. Neutral** ——> **Go to question # 42**
41. What are the reasons for not being in favor of vaccination?	1. Absence of beneficial effect<br>2. Presence of harmful effect<br>3. Others: ________________________________
42. Can you name an event in the past that diminished your trust in vaccination?	1.Yes<br>**2.No** ——> **Go to question # 44**

43. What was the nature of the event?	
44. What do you consider more important, vaccination of boys or girls?	1.Boys<br>2.Girls<br>3.Same importance
45. Do you search for information about immunization of children by yourself?	1.Yes<br>**2.No** ——> **Go to question #47**
46. If yes, how?	
47. How would you evaluate your knowledge about child immunization?	1.Good<br>2.Fair<br>3.Poor
48. Do you plan to have your child vaccinated with all the vaccines included in the Lebanese immunization program?	**1.Yes** ——> **Go to question # 50**<br>2.No
49. What are the reasons?	
50. Would you like to get more information about vaccination?	1.Yes<br>2.No
**D. Assessment of mother's practices towards immunization**	
51. Does your child have an immunization card?	1.Yes<br>2.No
52. Who is the decision maker (in matters of immunization of children)?	1. Father<br>2. Mother<br>3. Both<br>4. Others(specify):___________________
53. Does anyone help you with taking care of your child?	1. Yes<br>2. **No** ——> **Go to question #55**
54. Specify please:	1. Mother<br>2. Father<br>3. Brother<br>4. Sister<br>5. Relative<br>6. Friend<br>7. Others: _____________________

Appendix 1 – Questionnaire

55. What is your source of information regarding the immunization program?(Choose all that apply)	1.Doctors<br>2.Nurses<br>3.Pharmacist<br>4.Awareness campaigns<br>5.Social media<br>6.Television<br>7.Radio<br>8.Friends<br>9.Family<br>10.Newspaper<br>11.Others (specify): ____________________________
56. What is the immunization status of your child?	1. Never immunized<br>**2. Partially immunized** ——> **Go to question # 58**<br>**3. Fully immunized** ——> **Go to question # 58**
57. Why didn't you vaccinate your child? (Choose all that apply)	1.Vaccines are not provided for free (expensive)<br>2.Fear of adverse events<br>3.Difficulty to access medical centers/vaccines<br>4.Problems in understanding the complex vaccination schedule<br>5.Doubts about vaccine importance<br>6.No barriers<br>7.Lack of vaccines at healthcare centers<br>8.Others (specify):____
58. When did you start vaccination for your child?	1. At birth<br>2. Other Time (specify): ____________________________<br>3. I can't remember
59. Were there any side effects noticed?	1. Yes<br>2. **No** ——> **Go to question # 64**
60. What were the side effects?	1.Fever<br>2.Pain at site of injection<br>3.Rash/Redness<br>4.Body weakness<br>5.Body swelling<br>6.Shivering<br>7.Headache<br>8.Diarrhea<br>9.Vomiting<br>10.Others (specify): ______________________________
61. What did you do?	1.Give extra fluids<br>2.Give paracetamol<br>3.Place a cold damp cloth over the affected area<br>4.Bathe the child in cool water<br>5. Overdress the child<br>6.Give antibiotics<br>7.Others (specify):_________________________
62. Did you inform the doctor/healthcare workers?	1. Yes<br>**2. No** ——> **Go to question # 63**
63. What was done?	1. Provide medicine for illness<br>2. Others (specify):_____________

64. Have you ever refused a vaccine as you considered it to include porcine or other animal derived ingredients (non-halal)?	1. Yes<br>2. No
65. What is the next immunization that your child must get?	1. Hepatitis B<br>2. Polio<br>3. Pertussis<br>4. Diphtheria<br>5. Tetanus<br>6. PCV<br>7. Rubella<br>8. Mumps<br>9. Measles<br>10.Others (specify): ______________<br>11.I don't know
66. What was the vaccine your child has received last?	1. Hepatitis B<br>2. Polio<br>3. Pertussis<br>4. Diphtheria<br>5. Tetanus<br>6. PCV<br>7. Rubella<br>8. Mumps<br>9. Measles<br>10.Others (specify): ______________<br>11.I don't know
Vaccination history of previous children (if any)	
67. Were previous children (if any) immunized?	1. Yes<br>2. No
68. Was their immunization complete?	**1. Yes** ——> **Go to question # 70**<br>2. No
69. Why?	1. Vaccines are not provided for free (expensive)<br>2. Fear of adverse events<br>3. Difficulty to access medical centers/vaccines<br>4. Problems in understanding the complex vaccination schedule<br>5. Doubts about vaccine importance<br>6. No barriers (immunization was complete)<br>7. Lack of vaccines at healthcare centers<br>8. Others (specify): ____
70. Do you have the vaccination card right now?	1. Yes<br>2. No

Appendix 1 – Questionnaire

Compulsory Vaccines

National Lebanese Vaccination Schedule

Age	Vaccine	Taken	Not Tak-en	Late	Not sure
At birth	Hepatitis B - First dose				
2 months	IPV – First dose				
	**Pentavalent vaccine (pertussis - diphtheria - hepatitis B - tetanus -Hib) – First dose**				
	- Pertussis				
	- Diphtheria				
	- Hepatitis B				
	- Tetanus				
	- Hib				
4 months	OPV – Second dose				
	**Pentavalent vaccine (pertussis - diphtheria - hepatitis B - tetanus -Hib) – Second dose**				
	- Pertussis				
	- Diphtheria				
	- Hepatitis B				
	- Tetanus				
	- Hib				
	PCV13 -First dose				
6 months	OPV – Third dose				
	**Pentavalent vaccine (pertussis - diphtheria - hepatitis B - tetanus -Hib) – Third dose**				
	- Pertussis				
	- Diphtheria				
	- Hepatitis B				

Appendix 1 – Questionnaire

	- Tetanus				
	- Hib				
	PCV13 - second dose				
9 months	Measles – zero dose				
12 months	**Measles, Mumps and Rubella (MMR) – First dose**				
	Measles				
	Mumps				
	Rubella				
	PCV13 – First booster				
18 months	OPV – First booster dose				
	**Pentavalent vaccine (pertussis - diphtheria - hepatitis B - tetanus -Hib) – First booster dose**				
	- Pertussis				
	- Diphtheria				
	- Hepatitis B				
	- Tetanus				
	- Hib				
	**Measles, Mumps and Rubella (MMR) – Second dose**				
	Measles				
	Mumps				
	Rubella				
4-5 years	OPV- second booster dose				
	DTP(diphtheria- tetanus- pertussis)				

Appendix 1 – Questionnaire

Additional Vaccines

Age	Vaccine	Taken	Not Taken	Late
2 months	Rotavirus– First dose			
4 months	Rotavirus– Second dose			
6 months	Rotavirus– Third dose			
9 months	Meningococcal– First dose			
12 months	Hepatitis A– First dose			
	Meningococcal– Second dose			
18 months	Hepatitis A– Second dose			
Annual vaccination (Between 6 and 24 months)	Influenza			
